# Supplementary material for: An inducible amphipathic α-helix mediates subcellular targeting and membrane binding of RPE65
Source: Life Sci Alliance. 2022 Oct 20;6(1):e202201546. doi: 10.26508/lsa.202201546 (PMC9585964; doi:10.26508/lsa.202201546)
Supplement: Supplementary file 1 [file LSA-2022-01546_TableS1.docx]

Table S1. Structural information of all available RPE65 protein. Crystal structures of bovine RPE65 were collected via x-ray diffraction.

| PDB ID | Structure | Resolution (Å) | Molecular Weight (kDa) | Missing Residues | Monomer/Dimer |
| --- | --- | --- | --- | --- | --- |
| 3KVC | Crystal structure of bovine RPE65 at 1.9 angstrom resolution | 1.9 | 61.04 | 108-126;198-200;261-271 | Dimer |
| 4F3D | Structure of RPE65: P65 crystal form grown in Fos-choline-10 | 2.5 | 61.04 | 110-123;268-271 | Dimer |
| 3FSN | Crystal structure of RPE65 at 2.14 angstrom resolution | 2.14 | 61.04 | 109-126 | Dimer |
| 4F2Z | Crystal structure of RPE65 in a lipid environment | 3 | 61.04 | 111-125 | Dimer |
| 4F3A | Structure of RPE65: P65 22 crystal form, iridium derivative | 2.6 | 61.04 | 110-124 | Monomer |
| 4F30 | Structure of RPE65: P65 crystal form grown in ammonium phosphate solution | 3.15 | 61.04 | 111-120 | Monomer |
| 5ULG | Crystal structure of RPE65 in complex with MB-008 and palmitate | 2.1 | 61.04 | 110-126;197-201;268-271 | Dimer |
| 5UL5 | Crystal structure of RPE65 in complex with MB-008 and palmitate | 2.2 | 61.04 | 110-124 | Dimer |
| 4RSC | Crystal structure of RPE65 in complex with emixustat and palmitate | 1.8 | 61.04 | 110-124;197-201;268-271 | Dimer |
| 4RSE | Crystal structure of RPE65 in complex with MB-001 and palmitate | 2.39 | 60.935 | 110-126;197-201 | Dimer |
| 4ZHK | Crystal structure of RPE65 in complex with MB-002 | 2.09 | 60.935 | 110-124;197-201 | Dimer |
| 4RYX | Crystal structure of RPE65 in complex with emixustat and palmitate, P6522 crystal form | 2 | 61.04 | 111-122;198-200;267-271 | Monomer |
| 7L0E | Crystal structure of bovine RPE65 in complex with gem-fluoro emixustat and palmitate | 1.9 | 61.04 | 110-124; 198-202 | Dimer |
| 4RYY | Crystal structure of RPE65 in complex with R-emixustat and palmitate | 2.3 | 60.935 | 110-126; 197-201 | Dimer |
| 4RYZ | Crystal structure of RPE65 in complex with S-emixustat and palmitate | 2.5 | 60.935 | 110-126; 197-201 | Dimer |
| 7K88 | Crystal structure of bovine RPE65 in complex with hexaethylene glycol monooctyl ether | 2.1 | 60.935 | 108-126;198-200;262-271 | Dimer |
| 7K89 | Crystal structure of bovine RPE65 in complex with 4-fluoro-emixustat and palmitate | 2.15 | 60.935 | 108-126;197-201;267-271 | Dimer |
| 78KG | Crystal structure of bovine RPE65 in complex with 4-fluoro-MB-004 and palmitate | 1.95 | 60.935 | 107-126;196-201;264-271 | Dimer |
