# Supplementary material for: An inducible amphipathic α-helix mediates subcellular targeting and membrane binding of RPE65
Source: Life Sci Alliance. 2022 Oct 20;6(1):e202201546. doi: 10.26508/lsa.202201546 (PMC9585964; doi:10.26508/lsa.202201546)
Supplement: Supplementary file 2 [file LSA-2022-01546_SdataF3.pdf]

Image ID: 0002528_04
Acquire Time: Sep 10, 2020 12:53:12 PM

Acquisition Information

#	Image ID	Acquire Time	Channels	Resolution	Intensities	Quality	Analysis	Image Name	Comment
1	0002528_04	Sep 10, 2020 12:53:12 PM	700	169um	Auto	high	Manual	0002528_04	

Image Display Values

Channel	Color	Minimum	Maximum	K
700	Gray Scale (Black on White)	0.180	19.2	0

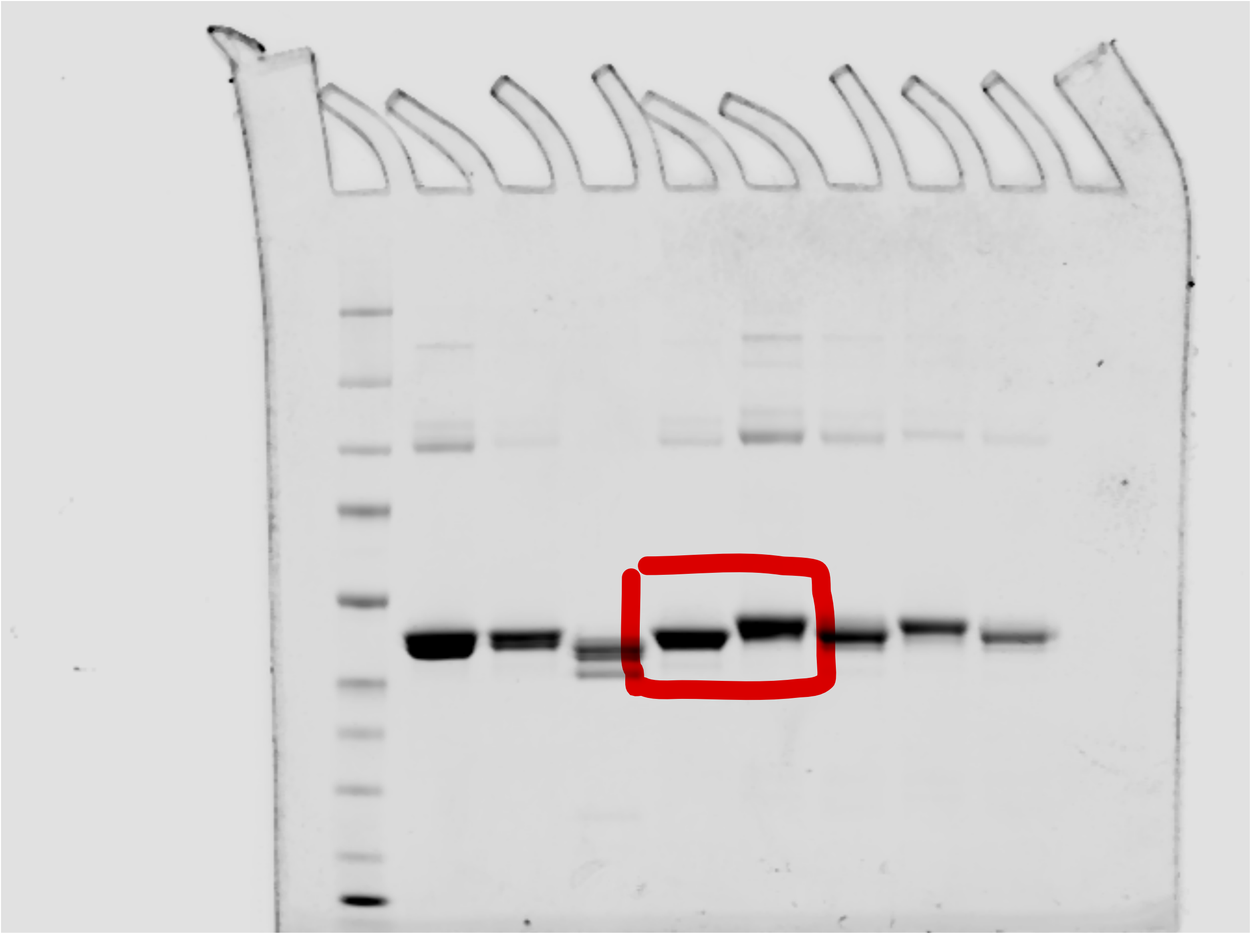

Acquisition Information (continued)

#	Image Modifications
1	Noise Removal Image ID: 0002528_01; Rotate 180 Image ID: 0002528_02; Flip Top to Bottom Image ID: 0002528_03
