# Supplementary figures and images for: An inducible amphipathic α-helix mediates subcellular targeting and membrane binding of RPE65
Source: Life Sci Alliance. 2022 Oct 20;6(1):e202201546. doi: 10.26508/lsa.202201546 (PMC9585964; doi:10.26508/lsa.202201546)

**
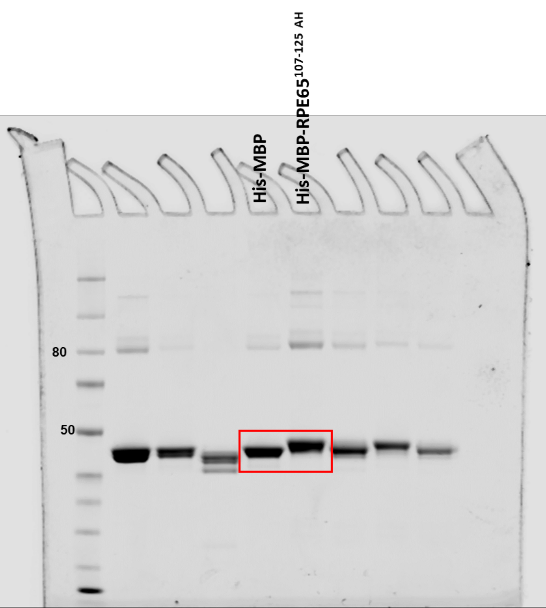
Raw Blots and Data**

**Fig. 3B**

**
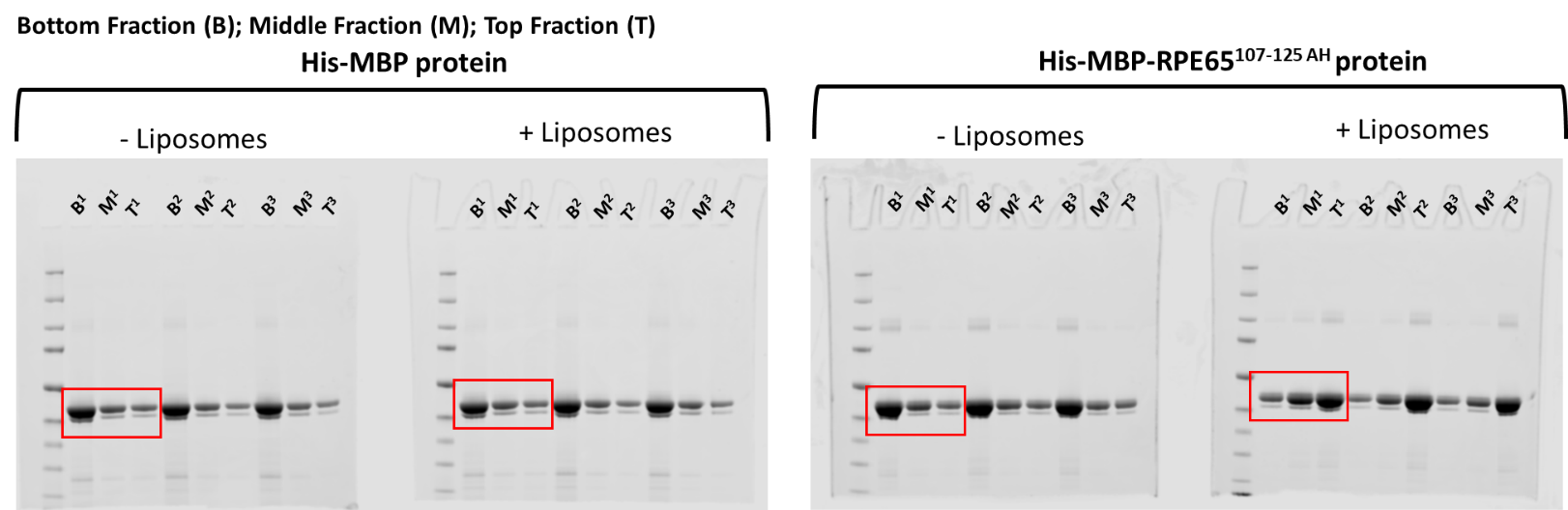
Fig. 3C**

**Fig. 5A**


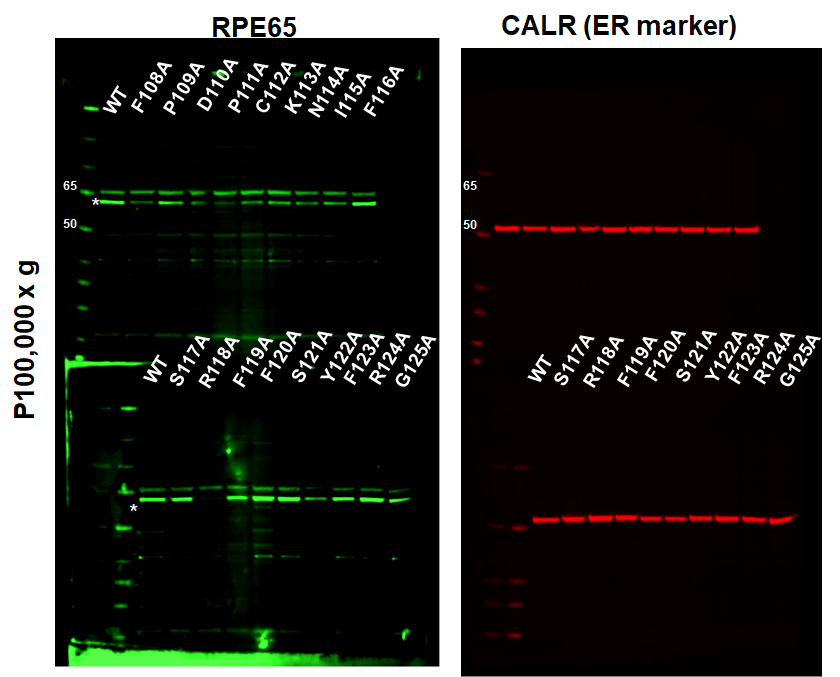

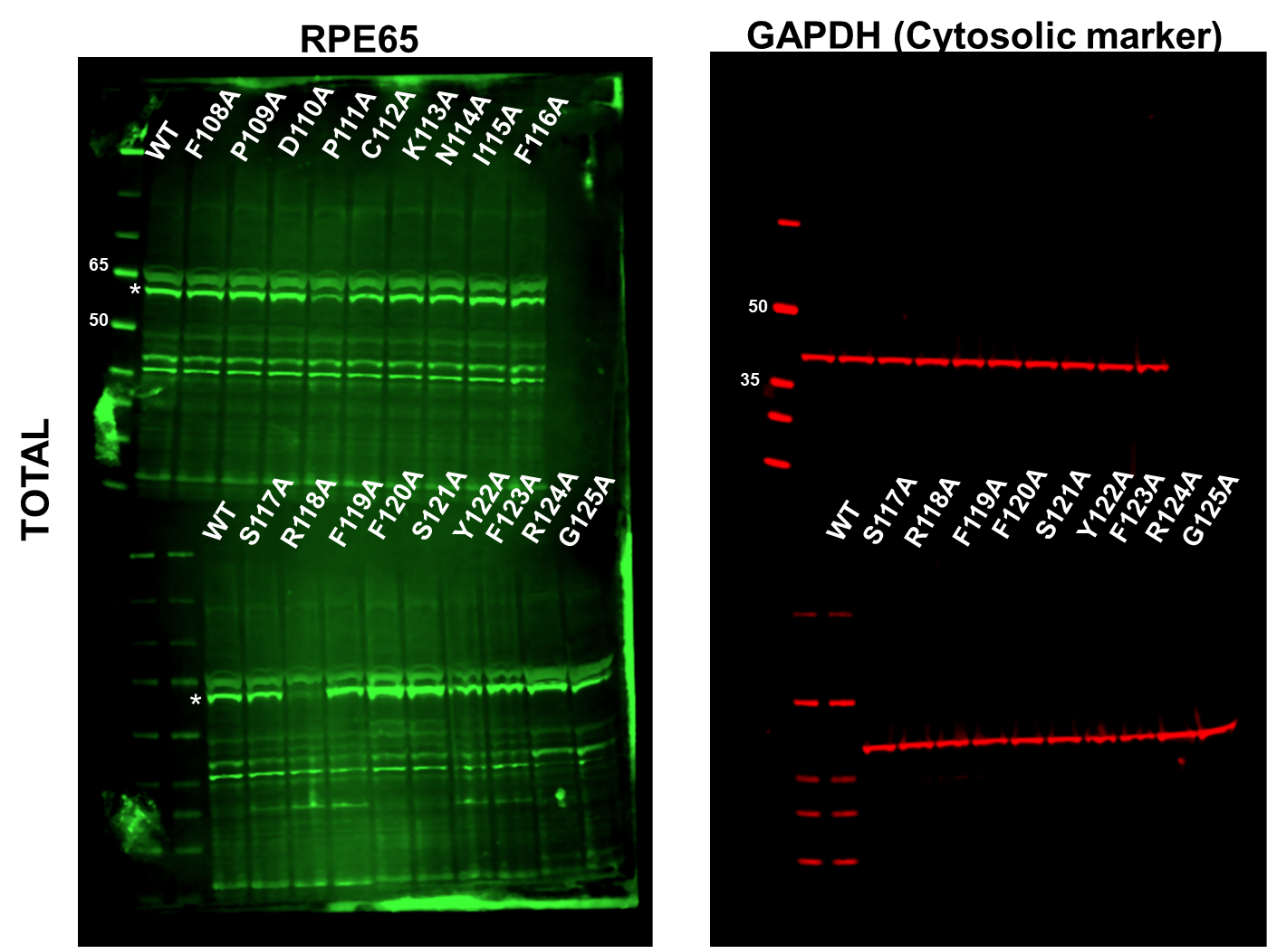


**Fig. 5B**


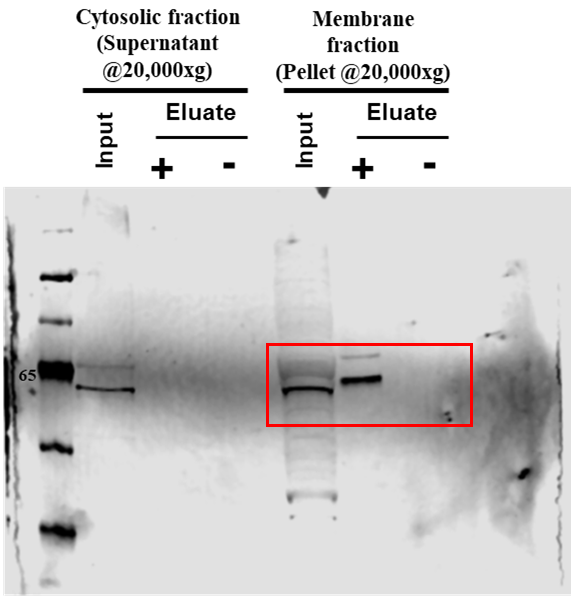


**WT**


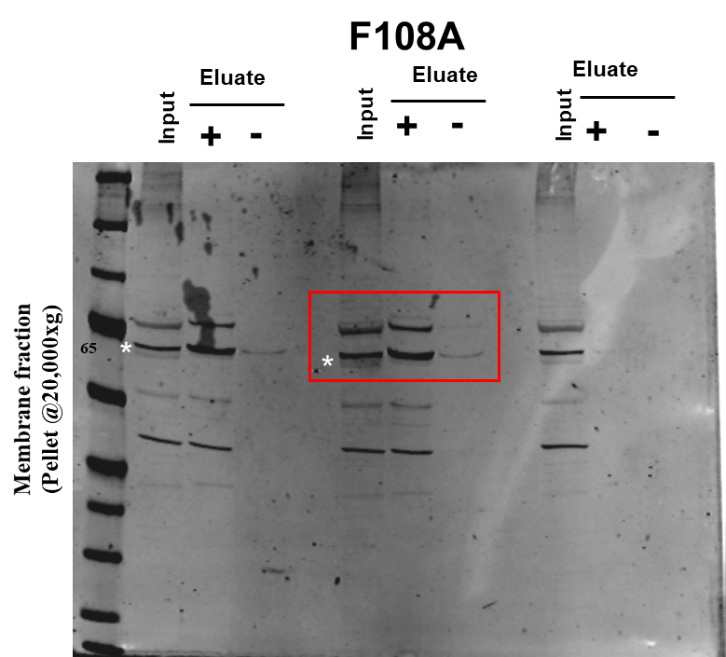

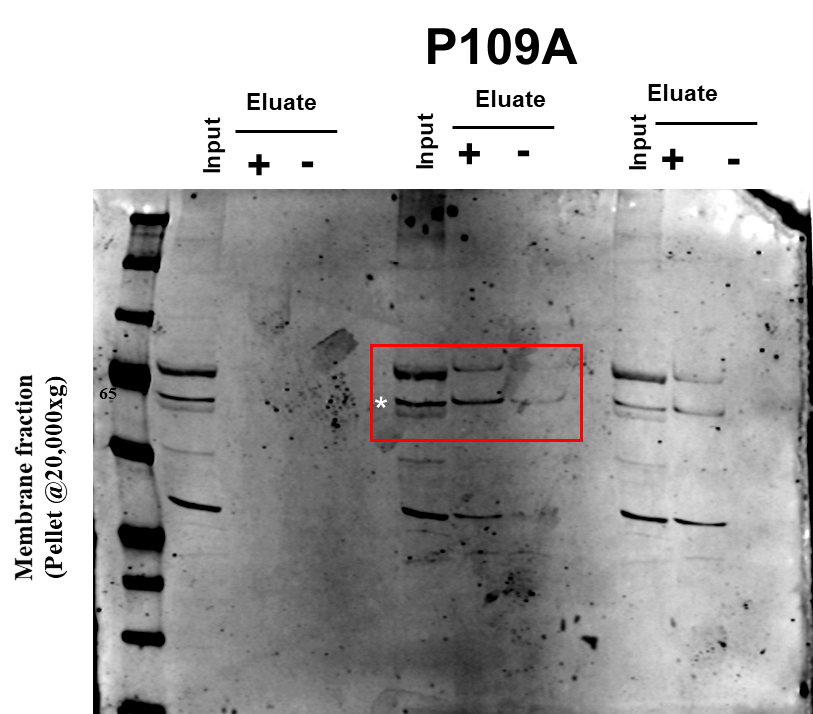

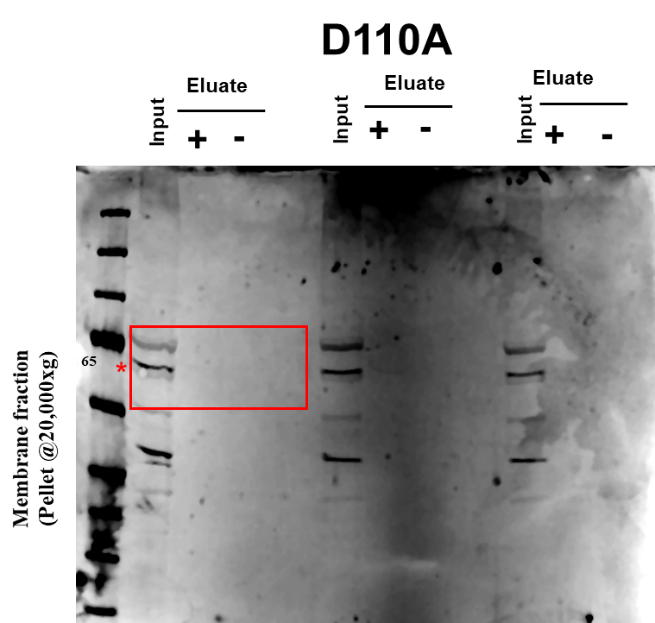

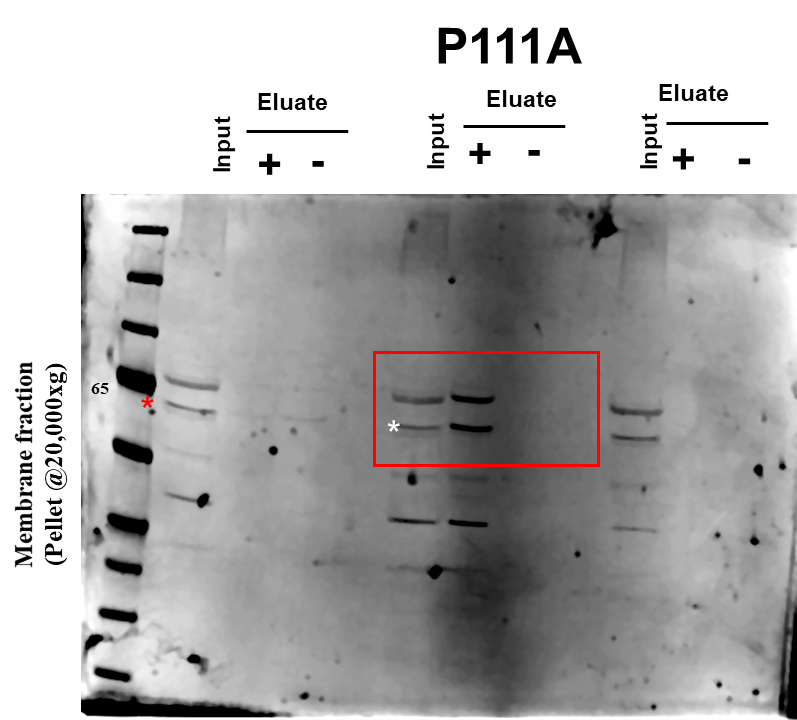

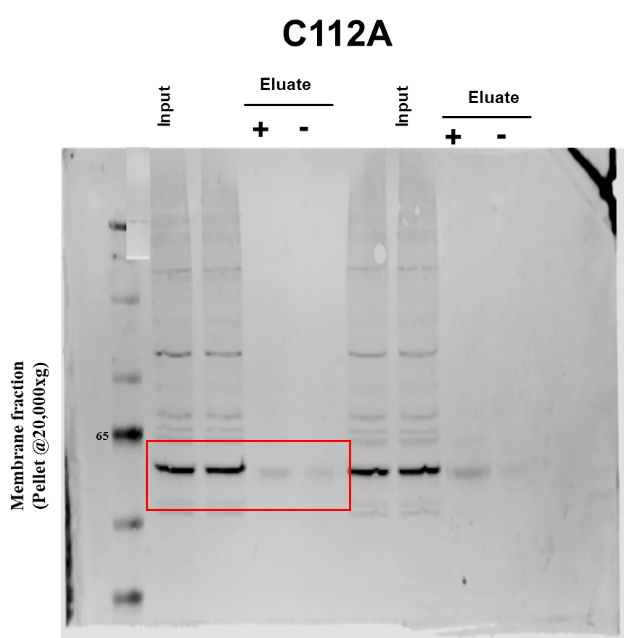

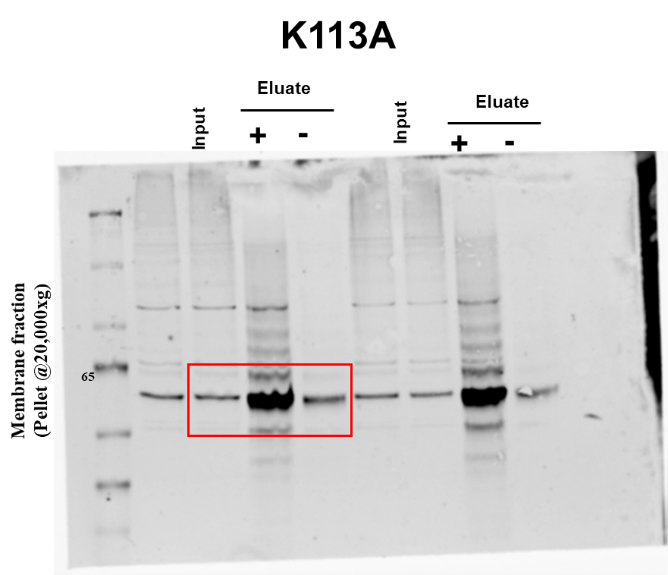

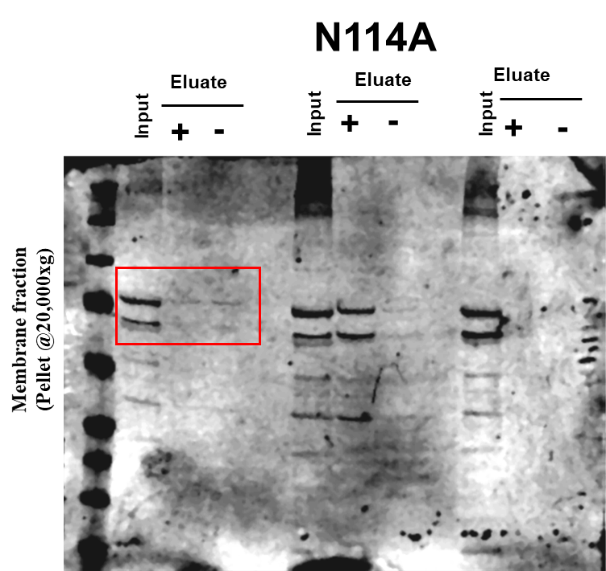

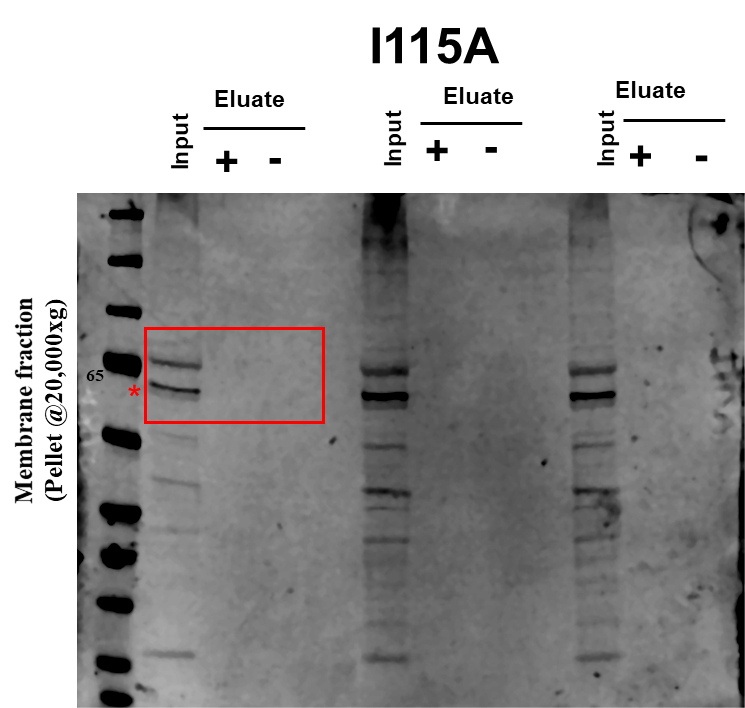

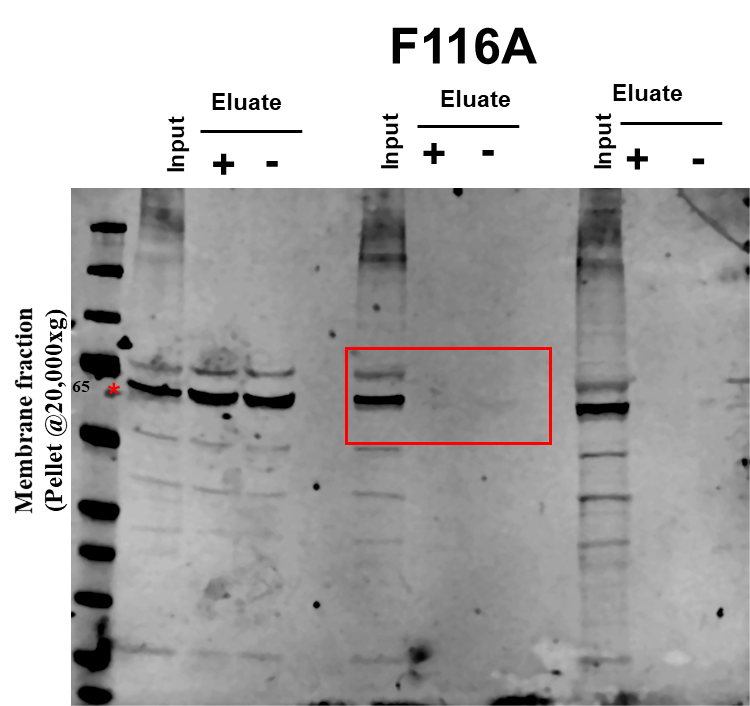

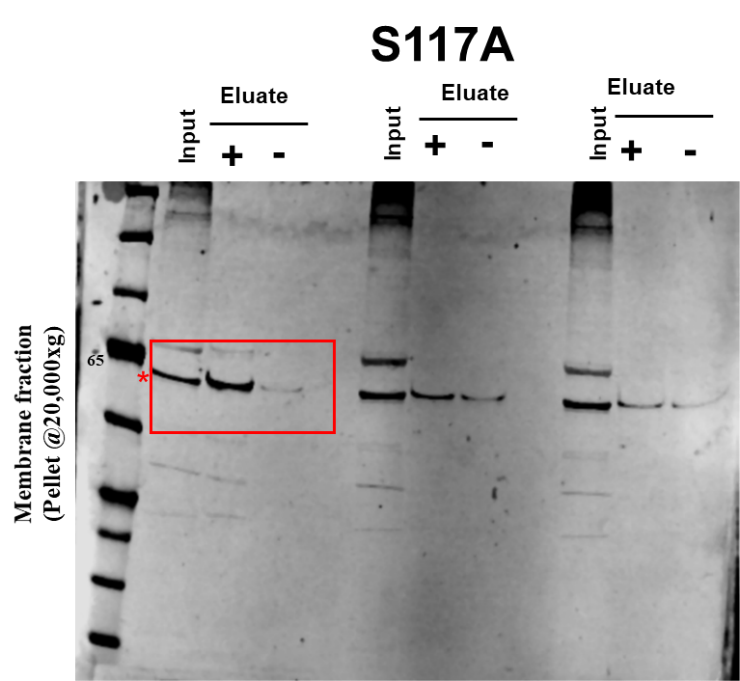

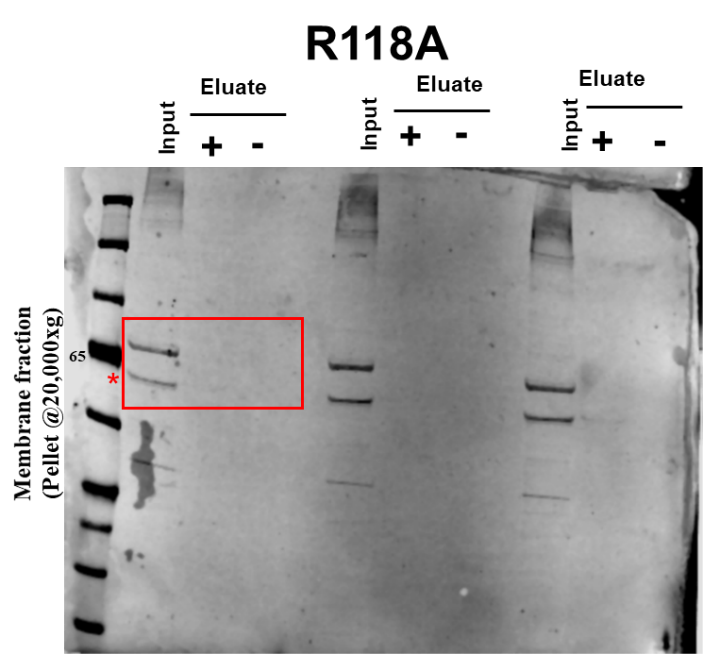

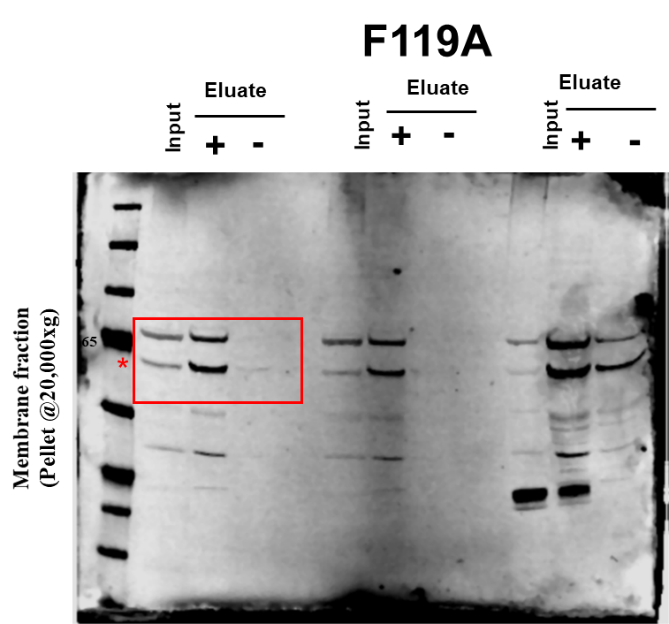

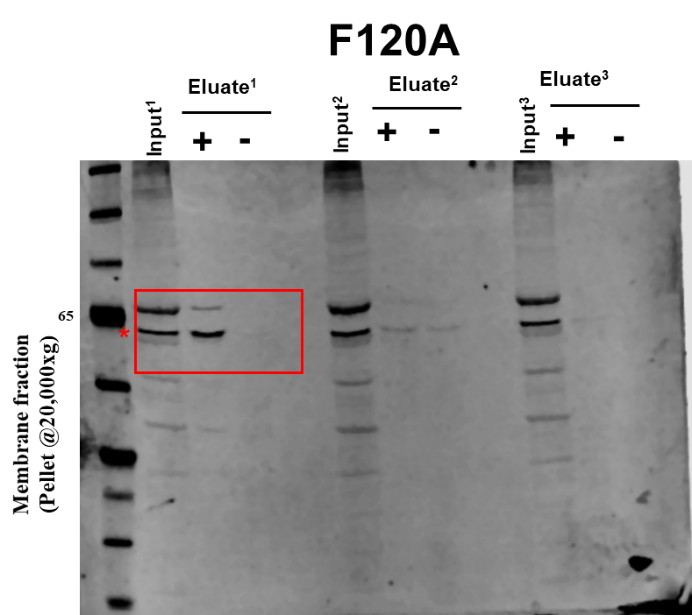

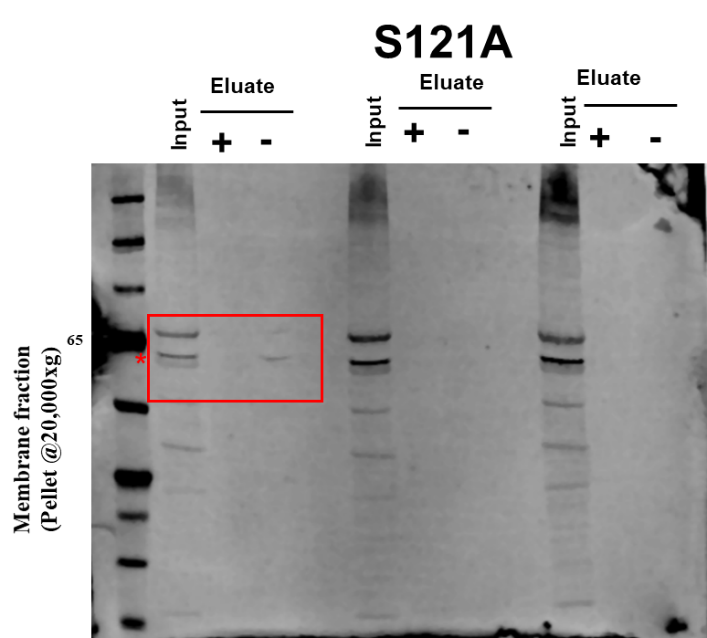

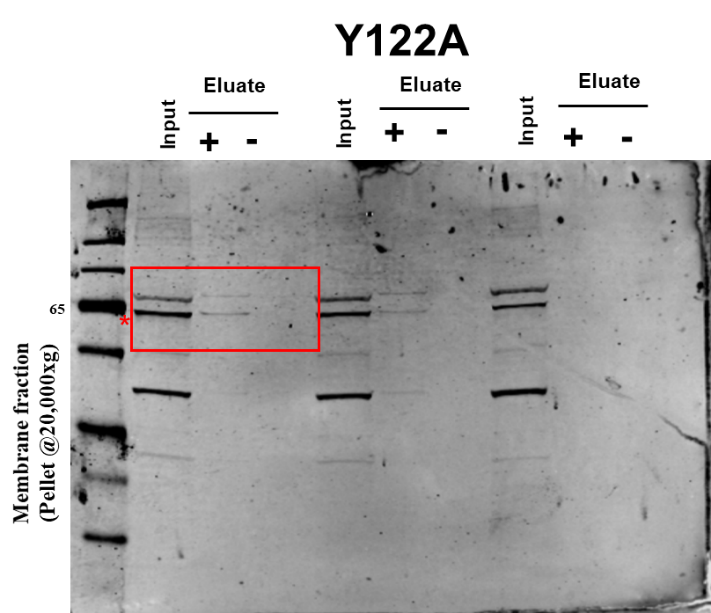

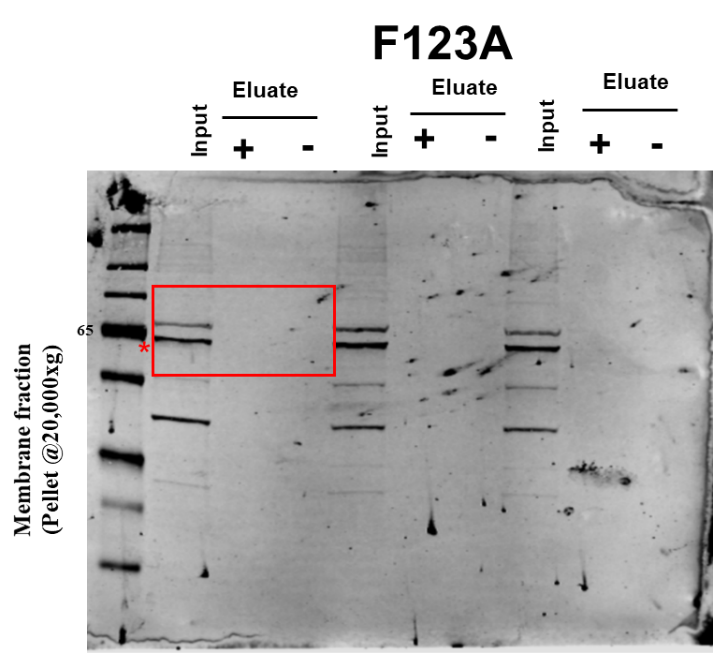

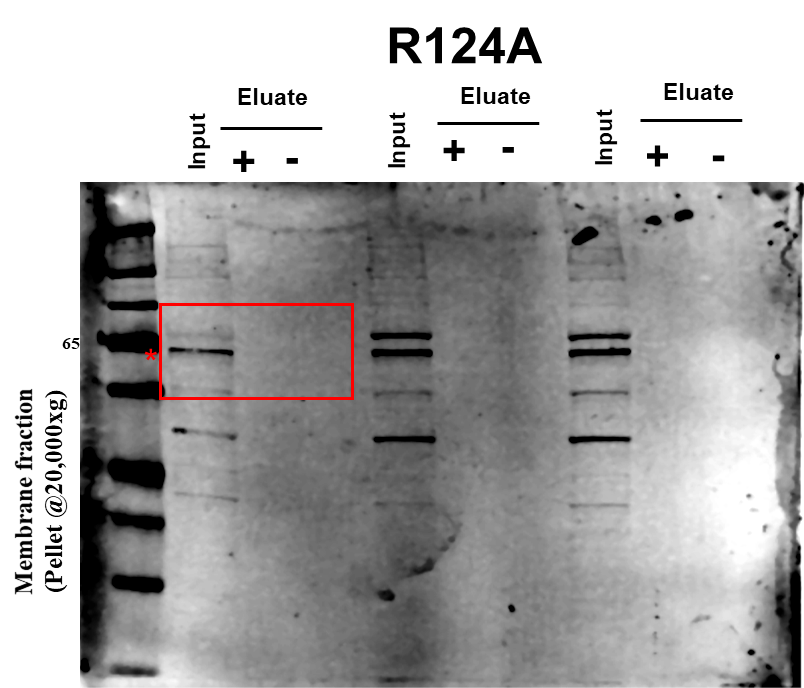


**
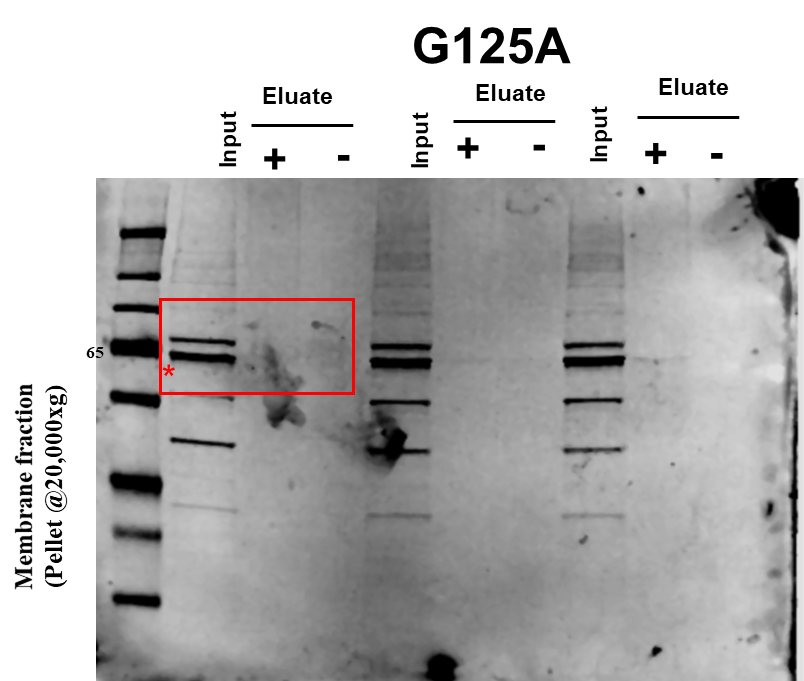
**

Supplement: Supplementary file 3 [file LSA-2022-01546_SdataF3_F5.docx]

Image Display Parameters

| Channel | Color                       | Minimum | Maximum | K |
|---------|-----------------------------|---------|---------|---|
| 800     | Gray Scale (Black on White) | 0.711   | 4.38    | 0 |

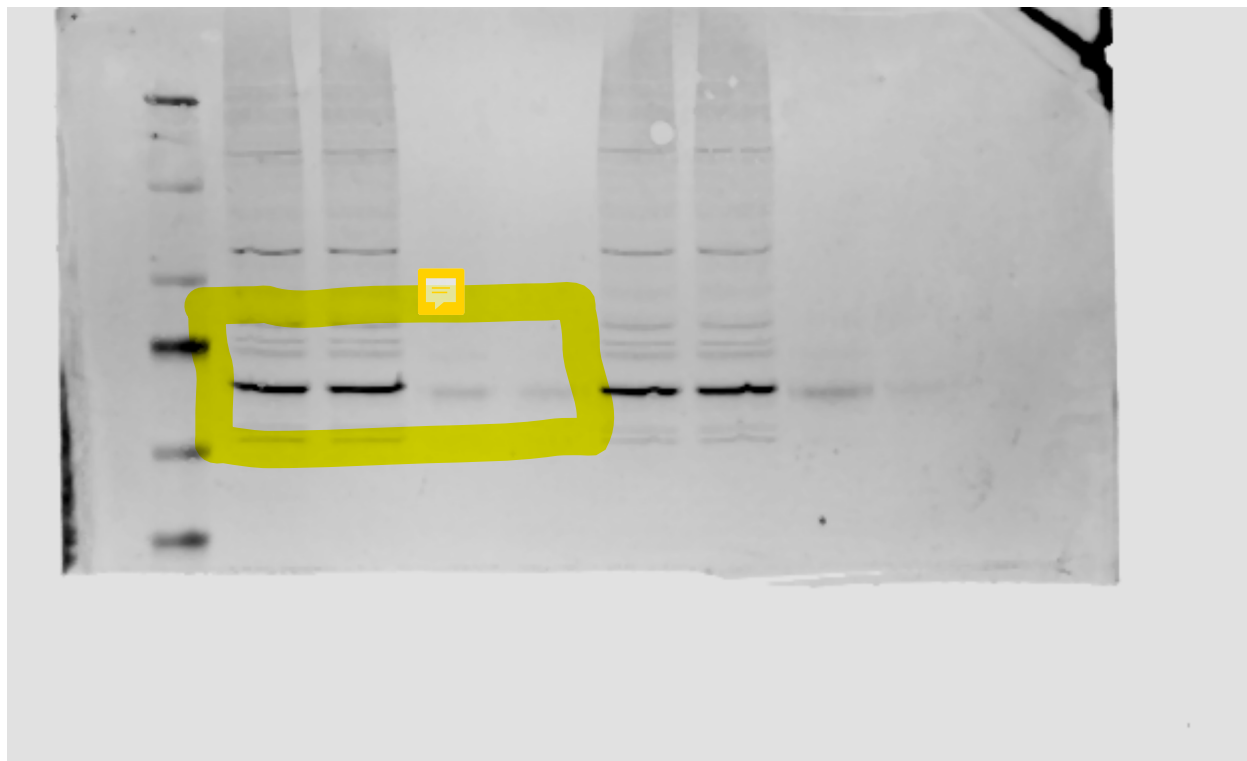

Supplement: Supplementary file 7 [file LSA-2022-01546_SdataF5.4.pdf]

Image Display Parameters

| Channel | Color                       | Minimum | Maximum | K |
|---------|-----------------------------|---------|---------|---|
| 800     | Gray Scale (Black on White) | 0.208   | 6.41    | 0 |

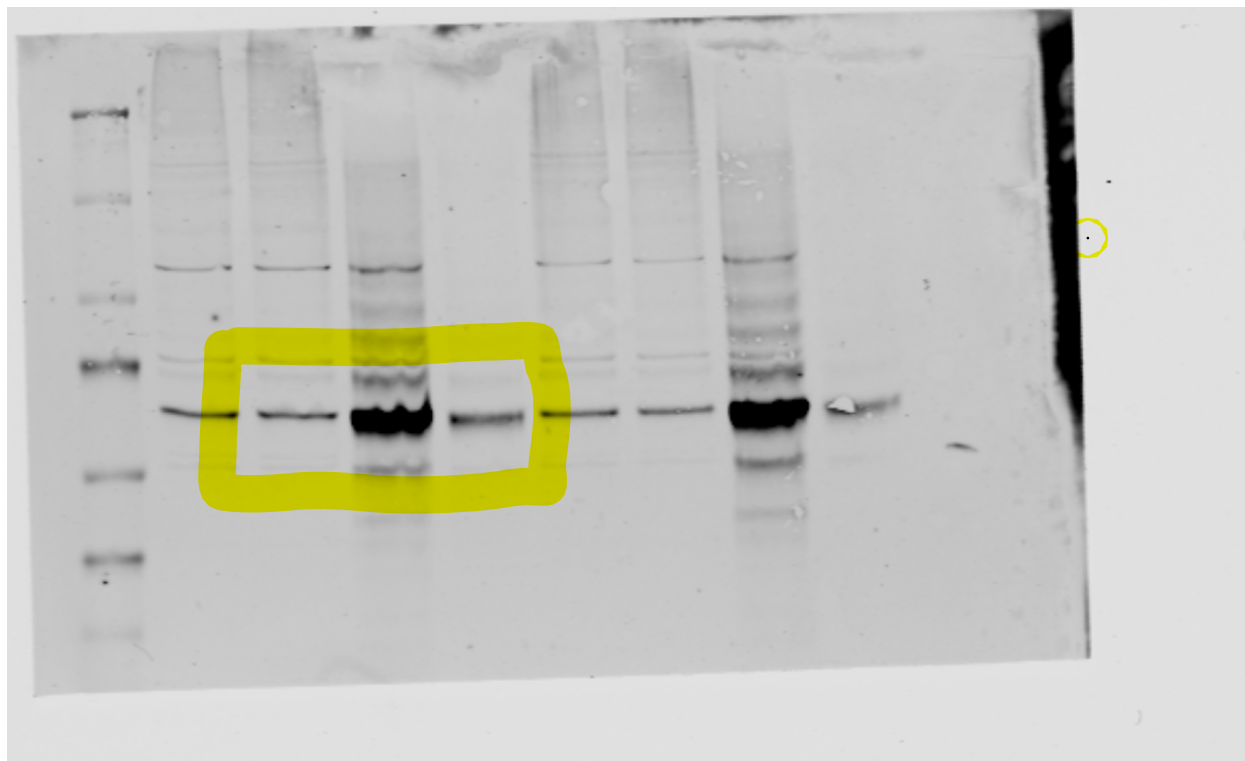

Supplement: Supplementary file 8 [file LSA-2022-01546_SdataF5.5.pdf]
