# Supplementary material for: An inducible amphipathic α-helix mediates subcellular targeting and membrane binding of RPE65
Source: Life Sci Alliance. 2022 Oct 20;6(1):e202201546. doi: 10.26508/lsa.202201546 (PMC9585964; doi:10.26508/lsa.202201546)
Supplement: Supplementary file 5 [file LSA-2022-01546_SdataF5.2.pdf]

Image ID: 0002441_01
Acquire Time: Aug 2, 2020 11:50:30 AM

Acquisition Information

#	Image ID	Acquire Time	Channels	Resolution	Intensities	Quality	Analysis	Image Name	Comment
1	0002441_01	Aug 2, 2020 11:50:30 AM	700 800	169um	Auto Auto	lowest	Manual	0002441_01	

Image Display Values

Channel	Color	Minimum	Maximum	K
800	Gray Scale (Black on White)	3.38	6.43	0

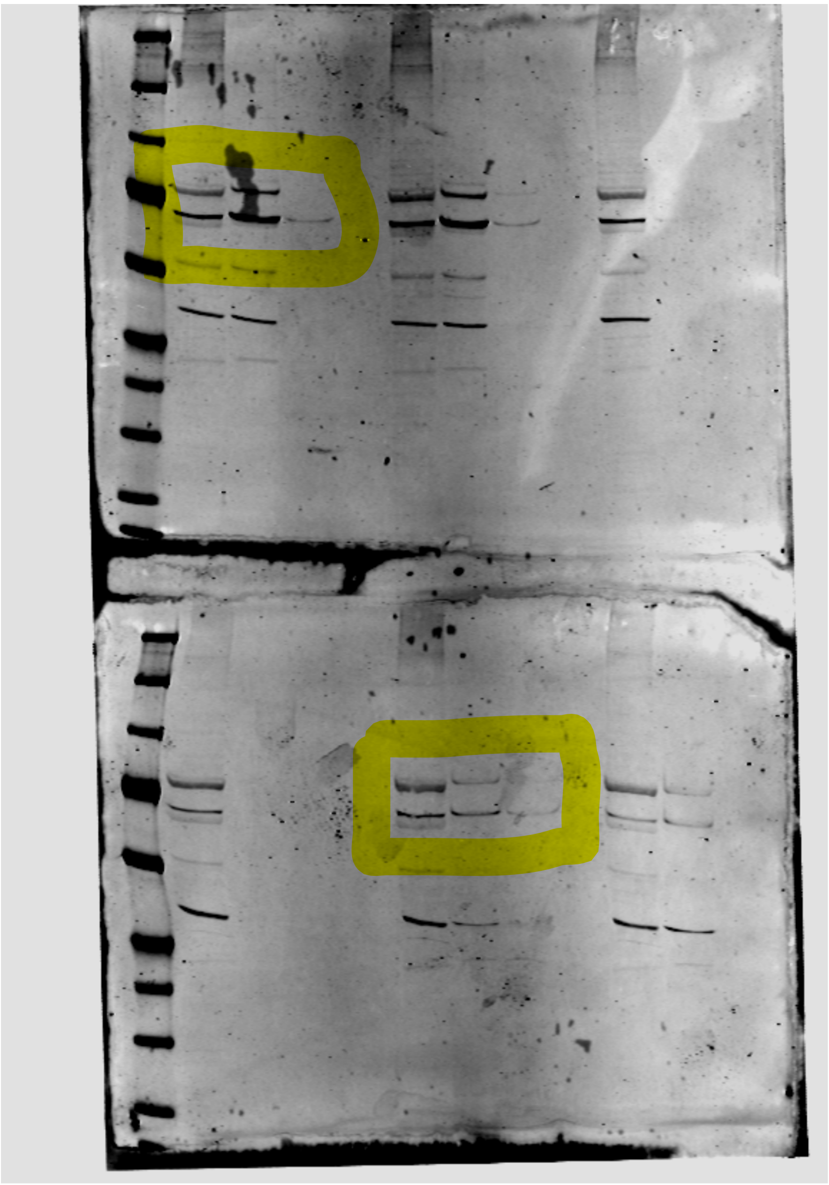

Acquisition Information (continued)

#	Image Modifications
1	
