# Supplementary material for: An inducible amphipathic α-helix mediates subcellular targeting and membrane binding of RPE65
Source: Life Sci Alliance. 2022 Oct 20;6(1):e202201546. doi: 10.26508/lsa.202201546 (PMC9585964; doi:10.26508/lsa.202201546)
Supplement: Supplementary file 9 [file LSA-2022-01546_SdataF5.6.pdf]

Image ID: 0002463_04
Acquire Time: Aug 7, 2020 12:53:18 PM

Acquisition Information

#	Image ID	Acquire Time	Channels	Resolution	Intensities	Quality	Analysis	Image Name	Comment
1	0002463_04	Aug 7, 2020 12:53:18 PM	700 800	169um	Auto Auto	low	Manual	0002463_04	

Image Display Values

Channel	Color	Minimum	Maximum	K
800	Gray Scale (Black on White)	1.03	1.84	0

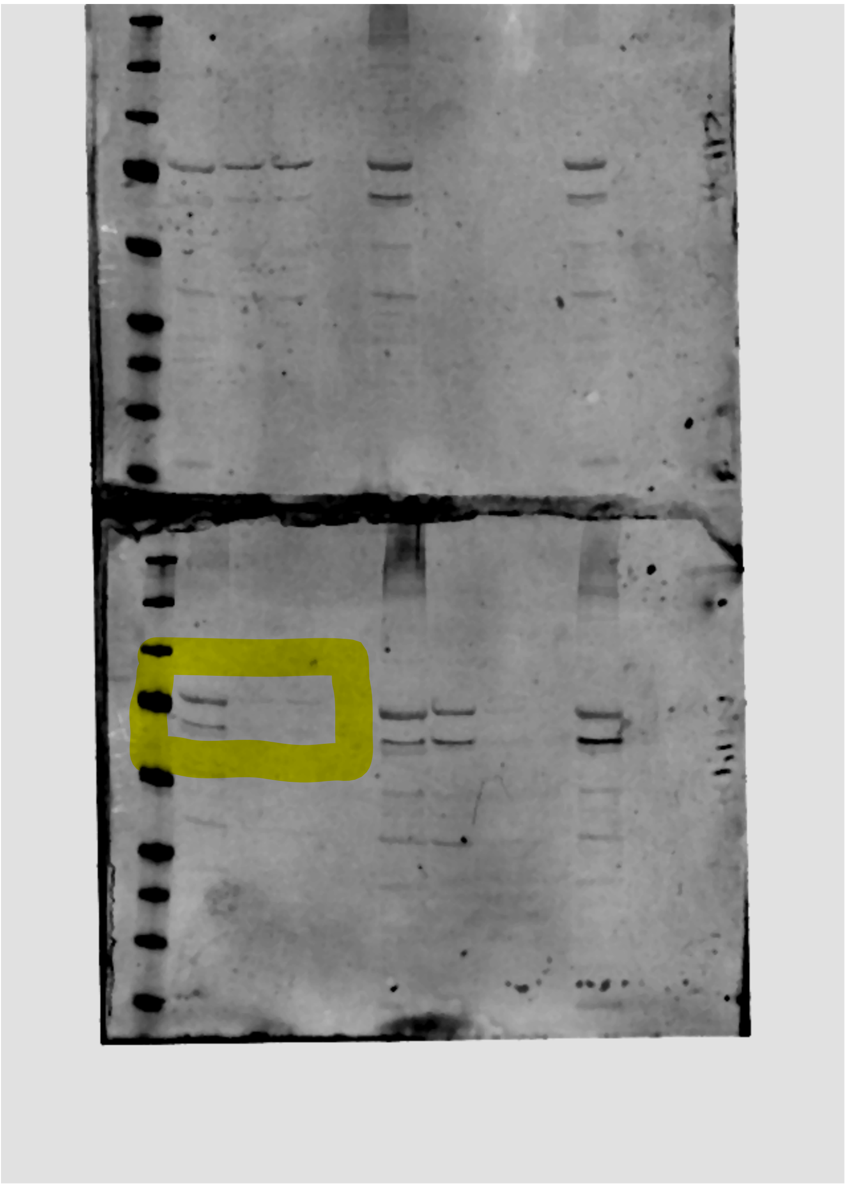

Acquisition Information (continued)

#	Image Modifications
1	Noise Removal Image ID: 0002463_01; Noise Removal Image ID: 0002463_02; Noise Removal Image ID: 0002463_03
