# Supplementary material for: An inducible amphipathic α-helix mediates subcellular targeting and membrane binding of RPE65
Source: Life Sci Alliance. 2022 Oct 20;6(1):e202201546. doi: 10.26508/lsa.202201546 (PMC9585964; doi:10.26508/lsa.202201546)
Supplement: Supplementary file 10 [file LSA-2022-01546_SdataF5.7.pdf]

Image ID: 0002464_04
Acquire Time: Aug 7, 2020 1:17:24 PM

Acquisition Information

#	Image ID	Acquire Time	Channels	Resolution	Intensities	Quality	Analysis	Image Name	Comment
1	0002464_04	Aug 7, 2020 1:17:24 PM	700 800	169um	Auto Auto	low	Manual	0002464_04	

Image Display Values

Channel	Color	Minimum	Maximum	K
800	Gray Scale (Black on White)	0.950	2.65	0

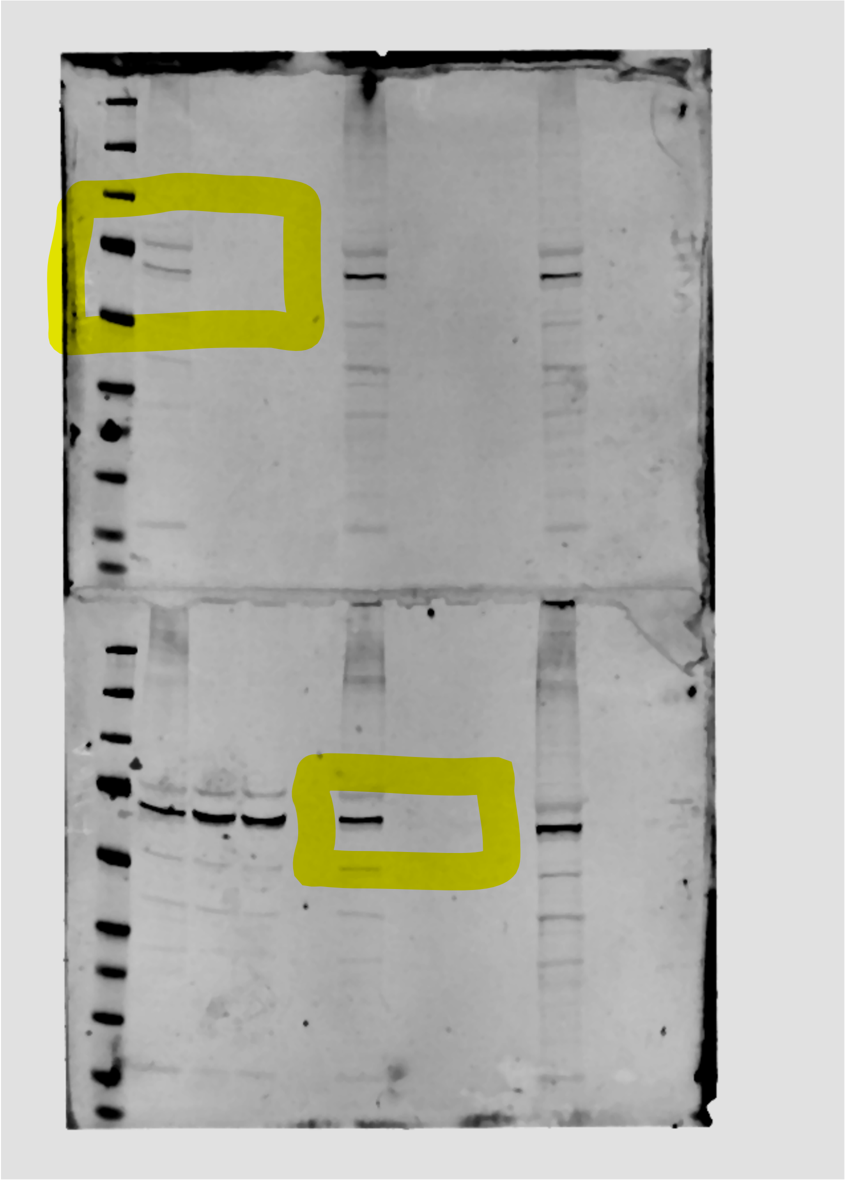

Acquisition Information (continued)

#	Image Modifications
1	Noise Removal Image ID: 0002464_01; Noise Removal Image ID: 0002464_02; Noise Removal Image ID: 0002464_03
