# Supplementary material for: An inducible amphipathic α-helix mediates subcellular targeting and membrane binding of RPE65
Source: Life Sci Alliance. 2022 Oct 20;6(1):e202201546. doi: 10.26508/lsa.202201546 (PMC9585964; doi:10.26508/lsa.202201546)
Supplement: Supplementary file 11 [file LSA-2022-01546_SdataF5.8.pdf]

LI-COR®

**Image ID: 0002474_02**
**Acquire Time: Aug 9, 2020 8:29:46 AM**

Acquisition Information

| # | Image ID | Acquire Time | Channels | Resolution | Intensities | Quality | Analysis | Image Name | Comment |
|---|---|---|---|---|---|---|---|---|---|
| 1 | 0002474_02 | Aug 9, 2020 8:29:46 AM | 700 800 | 169um | Auto Auto | low | Manual | 0002474_02 | |

Image Display Values

| Channel | Color | Minimum | Maximum | K |
|---|---|---|---|---|
| 800 | Gray Scale (Black on White) | 2.29 | 4.12 | 0 |

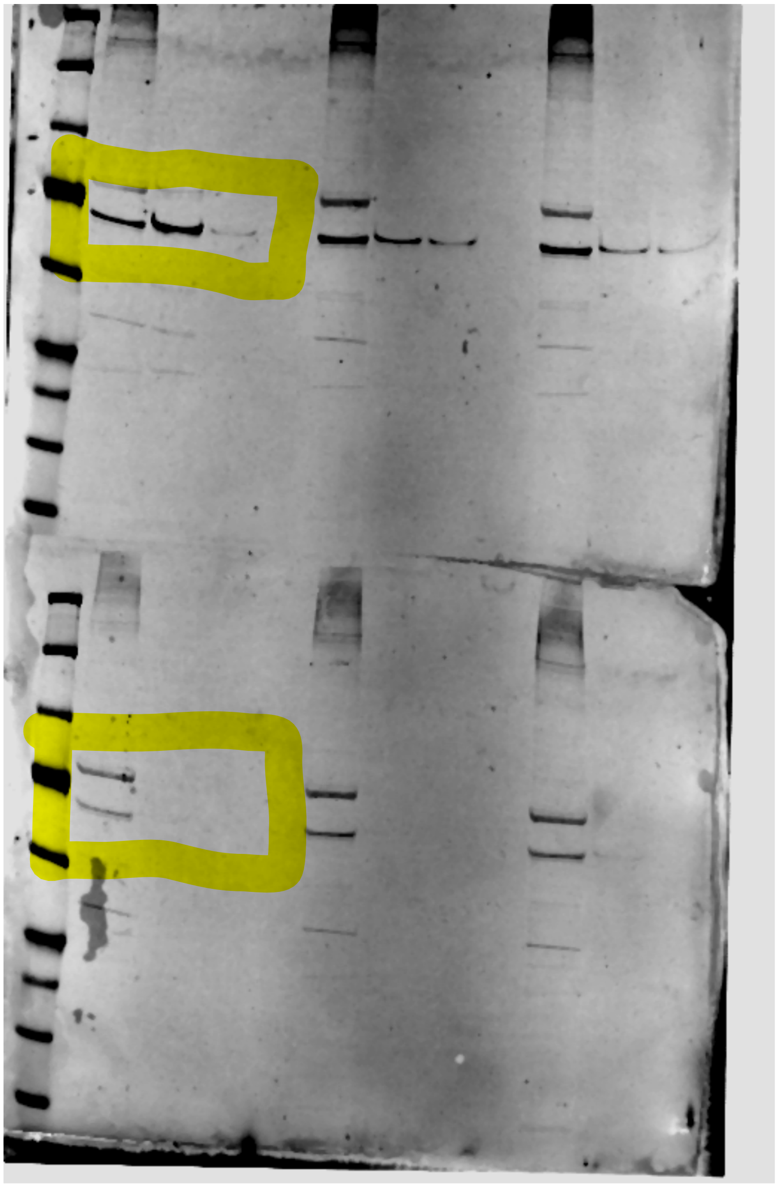

Acquisition Information (continued)

| # | Image Modifications |
|---|---|
| 1 | Noise Removal Image ID: 0002474_01 |
