# Supplementary material for: An inducible amphipathic α-helix mediates subcellular targeting and membrane binding of RPE65
Source: Life Sci Alliance. 2022 Oct 20;6(1):e202201546. doi: 10.26508/lsa.202201546 (PMC9585964; doi:10.26508/lsa.202201546)
Supplement: Supplementary file 16 [file LSA-2022-01546_TableS2.docx]

Table S2. RPE65 variants data were derived from https://databases.lovd.nl/shared/variants/RPE65

| **Exon/Intron** | **Change in the DNA (cDNA)** | **Change in the protein** | **Change in the protein function** | **Clinical classification** | **Reference** |
| --- | --- | --- | --- | --- | --- |
| - | c.329A>G | p.(Asp110Gly) | Affects  function | Likely pathogenic (recessive) | (Chung et al., 2019) |
| ***4*** | ***c.329A>T*** | ***p.(Asp110Val)*** | ***Affects***  ***function*** | ***Likely pathogenic (recessive)*** | (Henderson et al., 2007) |
| ***4-5*** | ***c.332C>A*** | ***p.(Pro111His)*** | ***Affects***  ***function*** | ***pathogenic (recessive)*** |  |
| - | c.335G>A | p.(Cys112Tyr) | Affects function | VUS | (Liu et al., 2021) |
| ***-*** | ***c.340A>C*** | ***p.(Asn114His)*** | ***Affects function*** | ***pathogenic (recessive)*** | (Zhong et al., 2019) |
| ***4*** | ***c.344T>C*** | ***p.(Ile115Thr)*** | ***Affects function*** | ***likely pathogenic (recessive)*** | (Schatz et al., 2011) |
| ***5*** | ***c.354G>T*** | ***p.(Arg118Ser)*** | ***Affects function*** | ***pathogenic (recessive)*** | (Stone, 2007) |
| ***5*** | ***c.361del*** | ***p.(Ser121Leufs*6),***  ***p.(Ser121Leufster6)*** | ***Affects function*** | ***pathogenic (recessive)*** | (Verma et al., 2013) |
| - | c.361delT | p.(Ser121Leufs*6) | Affects function | pathogenic | (Wang et al., 2014) |
| 5 | c.361dup | p.(Ser121Phefs*10) | Affects function | likely pathogenic (recessive) | (Wiszniewski et al., 2011) |
| - | c.361dupT | p.(Ser121Phefs*10) | Affects function | pathogenic |  |
| 5 | c.361_362insT | p.(Ser121Phefs*10) | Affects function | pathogenic (recessive) | (Verma et al., 2013) |
| - | c.364T>C | p.(Tyr122His) | Affects function | pathogenic (recessive) | (Li et al., 2020) |
| 5 | c.370C>T | p.(Arg124*) | Affects function | pathogenic (recessive) | (Pasadhika et al., 2010) |
