# Supplementary material for: An inducible amphipathic α-helix mediates subcellular targeting and membrane binding of RPE65
Source: Life Sci Alliance. 2022 Oct 20;6(1):e202201546. doi: 10.26508/lsa.202201546 (PMC9585964; doi:10.26508/lsa.202201546)
Supplement: Supplementary file 18 [file LSA-2022-01546_TableS3.docx]

Table S3. List of primers used in the study.

| F108AF | 5'- CTTGCAGGGATCTGGGGCCGCACAGGTGCCAAAT -3’ |
| --- | --- |
| F108AR | 5'- ATTTGGCACCTGTGCGGCCCCAGATCCCTGCAAG -3’ |
| P109AF | 5'- CTTGCAGGGATCTGCGAACGCACAGGTGC -3’ |
| P109AR | 5'- GCACCTGTGCGTTCGCAGATCCCTGCAAG -3' |
| D110AF | 5'- ATATATTCTTGCAGGGAGCTGGGAACGCACAGGTG -3' |
| D110AR | 5'- CACCTGTGCGTTCCCAGCTCCCTGCAAGAATATAT -3' |
| P111AF | 5'- AATATATTCTTGCAGGCATCTGGGAACGCACAGGT -3' |
| P111AR | 5'- ACCTGTGCGTTCCCAGATGCCTGCAAGAATATATT -3' |
| C112AF | 5'- ACCTGGAAAATATATTCTTGGCGGGATCTGGGAACGCACAG -3' |
| C112AR | 5'- CTGTGCGTTCCCAGATCCCGCCAAGAATATATTTTCCAGGT -3' |
| K113AF | 5'- AAAAACCTGGAAAATATATTCGCGCAGGGATCTGGGAACGCAC -3' |
| K113AR | 5'- GTGCGTTCCCAGATCCCTGCGCGAATATATTTTCCAGGTTTTT -3' |
| N114AF | 5'- GAAAAAAACCTGGAAAATATAGCCTTGCAGGGATCTGGGAACGC -3' |
| N114AR | 5'- GCGTTCCCAGATCCCTGCAAGGCTATATTTTCCAGGTTTTTTTC -3' |
| I115AF | 5'- AGTAAGAAAAAAACCTGGAAAATGCATTCTTGCAGGGATCTGGGAACG -3' |
| I115AR | 5'- CGTTCCCAGATCCCTGCAAGAATGCATTTTCCAGGTTTTTTTCTTACT -3' |
| F116AF | 5'- GAAAAAAACCTGGAAGCTATATTCTTGCAGGGATCTGGGAACGC -3' |
| F116AR | 5'- GCGTTCCCAGATCCCTGCAAGAATATAGCTTCCAGGTTTTTTTC -3' |
| S117AF | 5'- GTAAGAAAAAAACCTGGCAAATATATTCTTGCAGGGATCTGGGAAC -3' |
| S117AR | 5'- GTTCCCAGATCCCTGCAAGAATATATTTGCCAGGTTTTTTTCTTAC -3' |
| R118AF | 5'- CCCTCGGAAGTAAGAAAAAAACGCGGAAAATATATTCTTGCAGGGA -3' |
| R118AR | 5'- TCCCTGCAAGAATATATTTTCCGCGTTTTTTTCTTACTTCCGAGGG -3' |
| F119AF | 5'- CACCCCTCGGAAGTAAGAAAAAGCCCTGGAAAATATATTCTTGCAG -3’ |
| F119AR | 5'- CTGCAAGAATATATTTTCCAGGGCTTTTTCTTACTTCCGAGGGGTG -3’ |
| F120AF | 5'- CCACCCCTCGGAAGTAAGAAGCAAACCTGGAAAATATATTCTTGC -3’ |
| F120AR | 5'- GCAAGAATATATTTTCCAGGTTTGCTTCTTACTTCCGAGGGGTGG -3’ |
| S121AF | 5'- CCACCCCTCGGAAGTAAGCAAAAAACCTGGAAAATATATTCTTG -3’ |
| S121AR | 5'- CAAGAATATATTTTCCAGGTTTTTTGCTTACTTCCGAGGGGTGG -3’ |
| Y122AF | 5'- ACCTCCACCCCTCGGAAGGCAGAAAAAAACCTGGAAAATATATTCT -3’ |
| Y122AR | 5'- AGAATATATTTTCCAGGTTTTTTTCTGCCTTCCGAGGGGTGGAGGT -3’ |
| F123AF | 5'-GACCTCCACCCCTCGGGCGTAAGAAAAAAACCTGGAAAATATA -3’ |
| F123AR | 5'- TATATTTTCCAGGTTTTTTTCTTACGCCCGAGGGGTGGAGGTC -3’ |
| R124AF | 5'- GTGACCTCCACCCCTGCGAAGTAAGAAAAAAACCTGGAA -3’ |
| R124AR | 5'- TTCCAGGTTTTTTTCTTACTTCGCAGGGGTGGAGGTCAC -3’ |
| G125AF | 5’- GTGACCTCCACCGCTCGGAAGTAAGAAAAAAACCTGGAA -3’ |
| G125AR | 5’- TTCCAGGTTTTTTTCTTACTTCCGAGCGGTGGAGGTCAC -3’ |
| D110VF | 5’- ATATATTCTTGCAGGGAACTGGGAACGCACAGGTG -3’ |
| D110VR | 5’- CACCTGTGCGTTCCCAGTTCCCTGCAAGAATATAT |
| P111HF | 5’- GTGCGTTCCCAGATCACTGCAAGAATATATTTTCC -3’ |
| P111HR | 5’- GGAAAATATATTCTTGCAGTGATCTGGGAACGCAC -3’ |
| N114HF | 5’- GATCCCTGCAAGCATATATTTTCCAGG -3’ |
| N114HR | 5’- CCTGGAAAATATATGCTTGCAGGGATC -3’ |
| I115TF | 5’- AAAAAAACCTGGAAAACGTATTCTTGCAGGGATCTGGGAACGC -3’ |
| I115TR | 5’- GCGTTCCCAGATCCCTGCAAGAATACGTTTTCCAGGTTTTTTTC -3’ |
| R118SF | 5’- CCCTCGGAAGTAAGAAAAAAAGCTGGAAAATATATTCTTGCAG -3’ |
| R118SR | 5’- CTGCAAGAATATATTTTCCAGCTTTTTTTCTTACTTCCGAGGG -3’ |
| S121LF | 5’- TCCAGGTTTTTTCTCTACTTCCGAGGG -3’ |
| S121LR | 5’- CCCTCGGAAGTAGAGAAAAAACCTGGA -3’ |
| 108_125RPE65Fwd | 5’- TACTTCCAATCCAATGCATTCCCAGATCCCTGCAAG -3’ |
| 108_125RPE65Rev | 5’- TTATCCACTTCCAATGTTATTACCCTCGGAAGTAAG-3’ |

Figure S1.


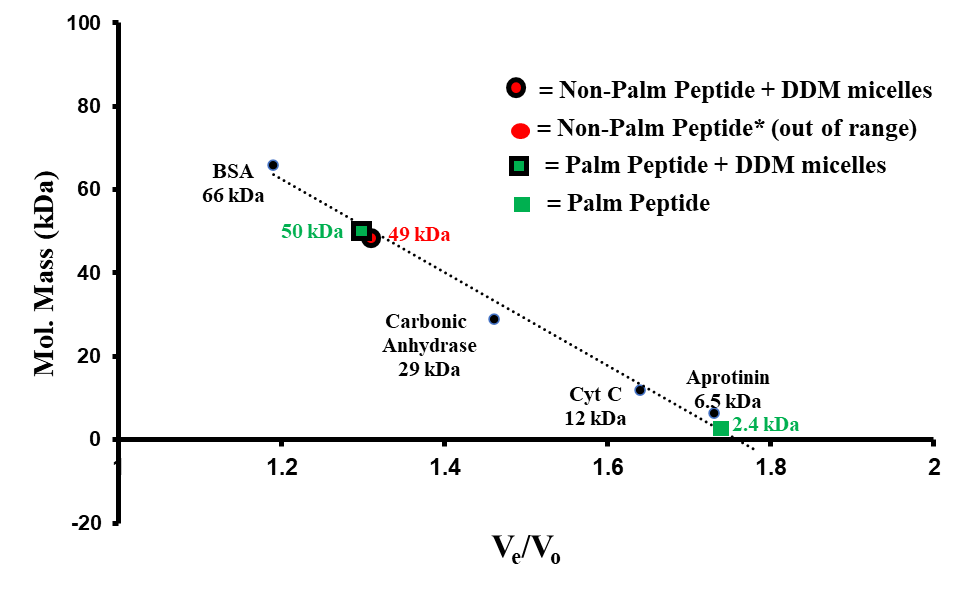


Figure S2. Pathogenic mutations/variants of unknown significance (VUS) in AH^107-125^ significantly impact the protein expression level of RPE65 protein.


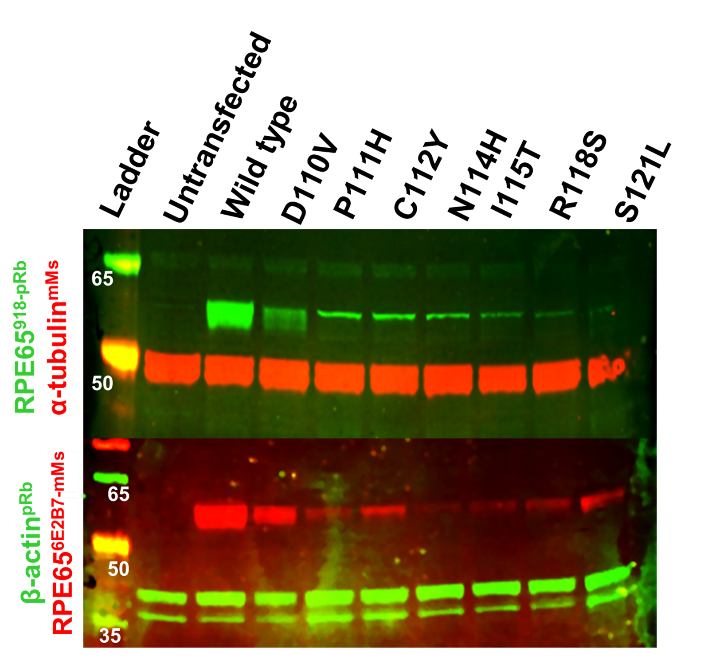

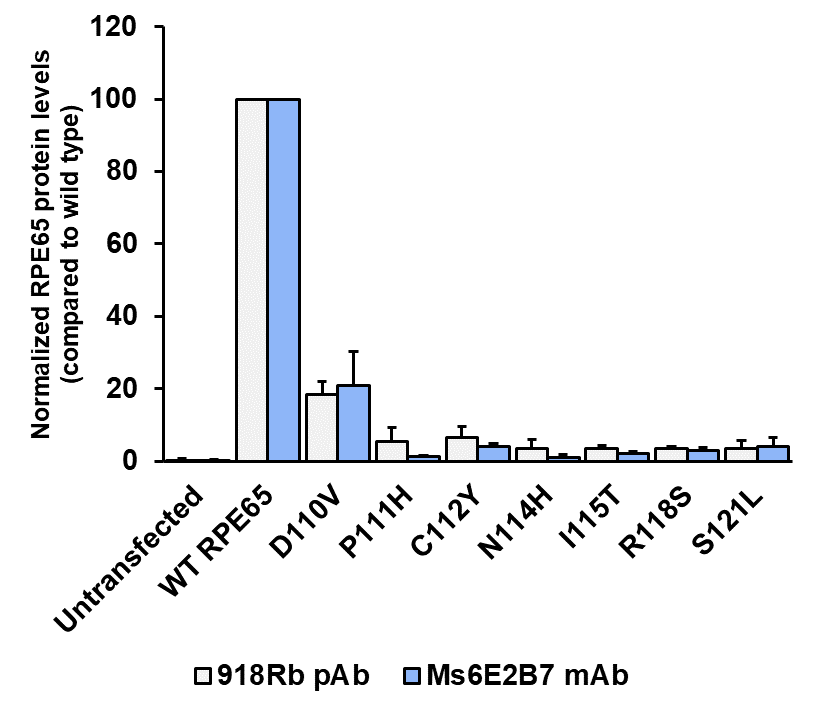


**References**

Chung, D.C., M. Bertelsen, B. Lorenz, M.E. Pennesi, B.P. Leroy, C.P. Hamel, E. Pierce, J. Sallum, M. Larsen, K. Stieger, M. Preising, R. Weleber, P. Yang, E. Place, E. Liu, G. Schaefer, J. DiStefano-Pappas, O.U. Elci, S. McCague, J.A. Wellman, K.A. High, and K.Z. Reape. 2019. The Natural History of Inherited Retinal Dystrophy Due to Biallelic Mutations in the RPE65 Gene. *Am J Ophthalmol*. 199:58-70.

Henderson, R.H., N. Waseem, R. Searle, J. van der Spuy, I. Russell-Eggitt, S.S. Bhattacharya, D.A. Thompson, G.E. Holder, M.E. Cheetham, A.R. Webster, and A.T. Moore. 2007. An assessment of the apex microarray technology in genotyping patients with Leber congenital amaurosis and early-onset severe retinal dystrophy. *Invest Ophthalmol Vis Sci*. 48:5684-5689.

Li, S., X. Xiao, Z. Yi, W. Sun, P. Wang, and Q. Zhang. 2020. RPE65 mutation frequency and phenotypic variation according to exome sequencing in a tertiary centre for genetic eye diseases in China. *Acta Ophthalmol*. 98:e181-e190.

Liu, X., T. Tao, L. Zhao, G. Li, and L. Yang. 2021. Molecular diagnosis based on comprehensive genetic testing in 800 Chinese families with non-syndromic inherited retinal dystrophies. *Clin Exp Ophthalmol*. 49:46-59.

Pasadhika, S., G.A. Fishman, E.M. Stone, M. Lindeman, R. Zelkha, I. Lopez, R.K. Koenekoop, and M. Shahidi. 2010. Differential macular morphology in patients with RPE65-, CEP290-, GUCY2D-, and AIPL1-related Leber congenital amaurosis. *Invest Ophthalmol Vis Sci*. 51:2608-2614.

Schatz, P., M. Preising, B. Lorenz, B. Sander, M. Larsen, and T. Rosenberg. 2011. Fundus albipunctatus associated with compound heterozygous mutations in RPE65. *Ophthalmology*. 118:888-894.

Stone, E.M. 2007. Leber congenital amaurosis - a model for efficient genetic testing of heterogeneous disorders: LXIV Edward Jackson Memorial Lecture. *Am J Ophthalmol*. 144:791-811.

Verma, A., V. Perumalsamy, S. Shetty, M. Kulm, and P. Sundaresan. 2013. Mutational screening of LCA genes emphasizing RPE65 in South Indian cohort of patients. *PLoS One*. 8:e73172.

Wang, J., V.W. Zhang, Y. Feng, X. Tian, F.Y. Li, C. Truong, G. Wang, P.W. Chiang, R.A. Lewis, and L.J. Wong. 2014. Dependable and efficient clinical utility of target capture-based deep sequencing in molecular diagnosis of retinitis pigmentosa. *Invest Ophthalmol Vis Sci*. 55:6213-6223.

Wiszniewski, W., R.A. Lewis, D.W. Stockton, J. Peng, G. Mardon, R. Chen, and J.R. Lupski. 2011. Potential involvement of more than one locus in trait manifestation for individuals with Leber congenital amaurosis. *Hum Genet*. 129:319-327.

Zhong, Z., F. Rong, Y. Dai, A. Yibulayin, L. Zeng, J. Liao, L. Wang, Z. Huang, Z. Zhou, and J. Chen. 2019. Seven novel variants expand the spectrum of RPE65-related Leber congenital amaurosis in the Chinese population. *Mol Vis*. 25:204-214.
